# Supplementary material for: Design and Synthesis of Some New Furan-Based Derivatives and Evaluation of In Vitro Cytotoxic Activity
Source: Molecules. 2022 Apr 18;27(8):2606. doi: 10.3390/molecules27082606 (PMC9024937; doi:10.3390/molecules27082606)
Supplement: Supplementary file 1 [file molecules-27-02606-s001.zip › molecules-1675027-supplementary.pdf]

# Supplementary Materials

# Design and Synthesis of Some New Furan-Based Derivatives and Evaluation of In Vitro Cytotoxic Activity

**Syed Nasir Abbas Bukhari [1,*], Hasan Ejaz [2], Mervat A. Elsherif [3], Kashaf Junaid [2], Islam Zaki [4,*] and Reham E. Masoud [5]**

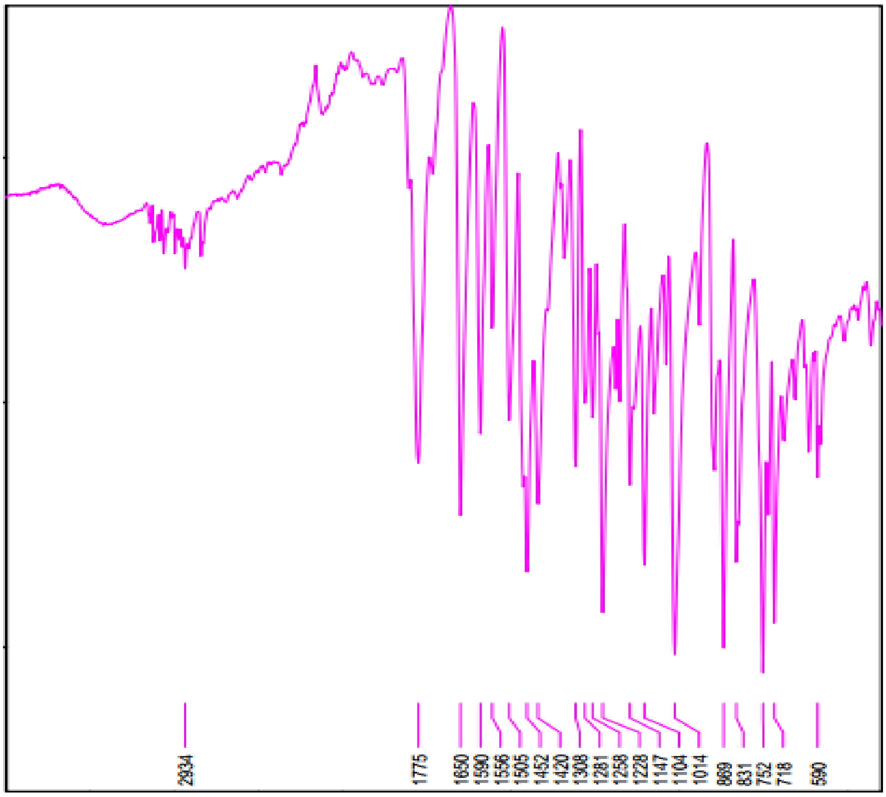


**Figure S1:** IR spectrum of compound **1.**

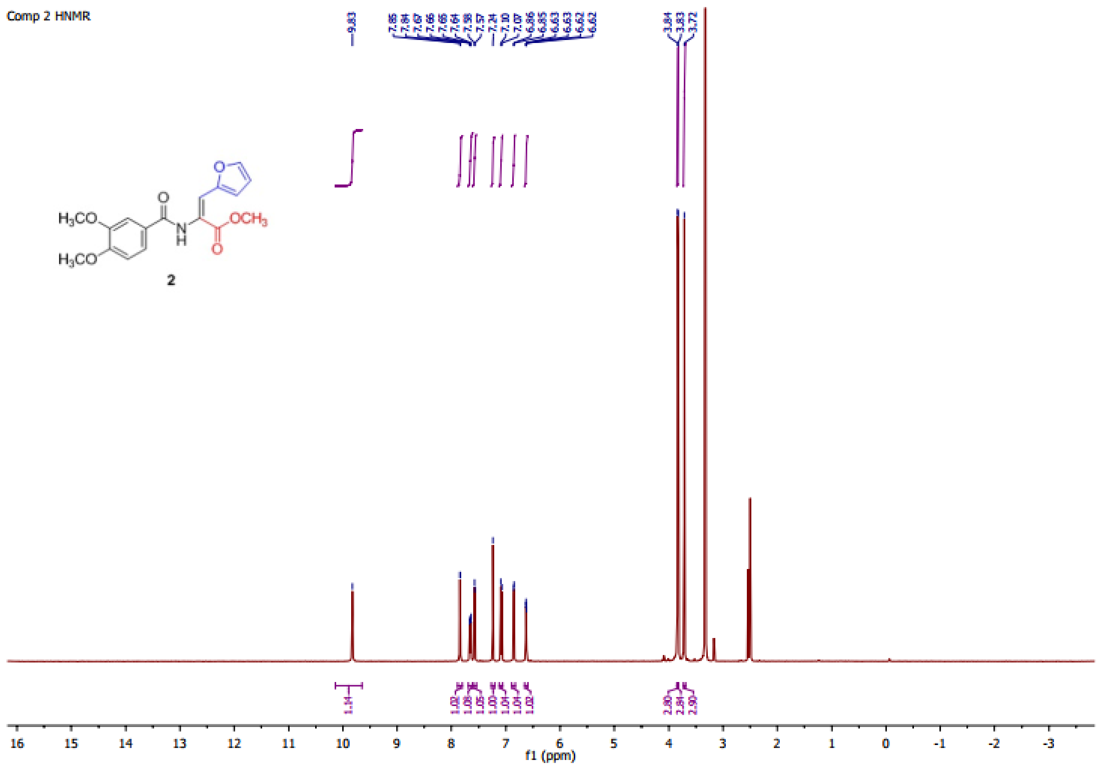


**Figure S2:** $^{1}$H-NMR spectrum of compound **2.**

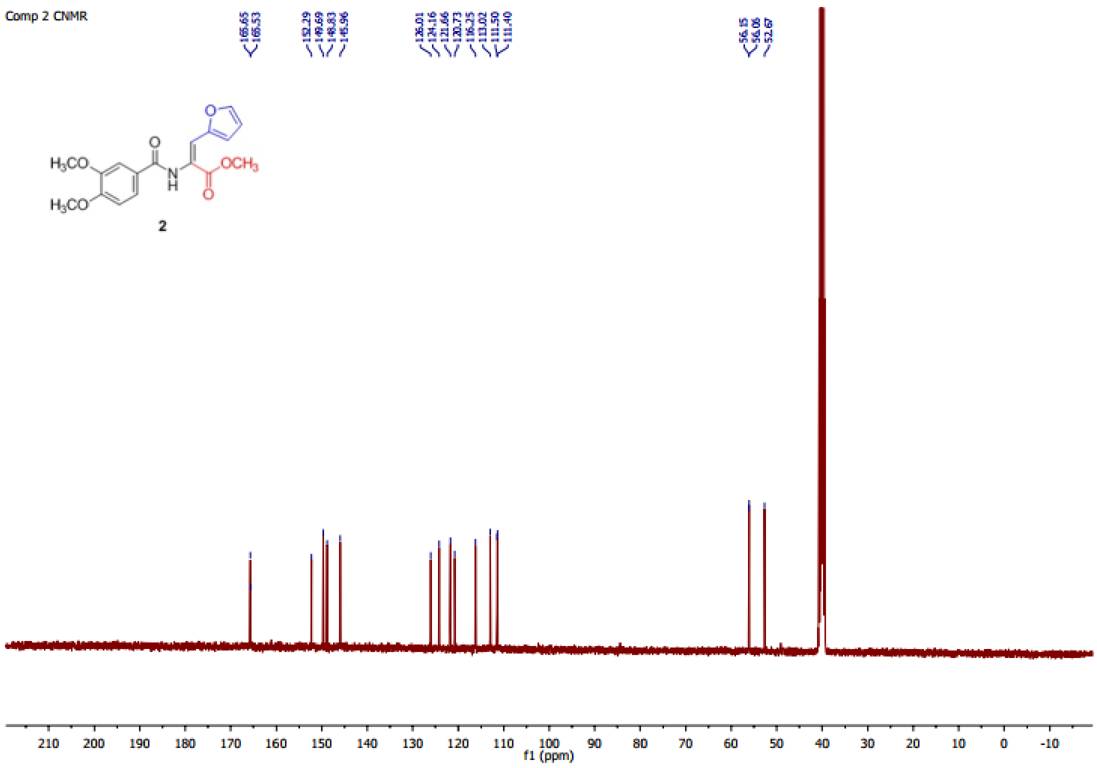


**Figure S3:** $^{13}$C-NMR spectrum of compound **2.**

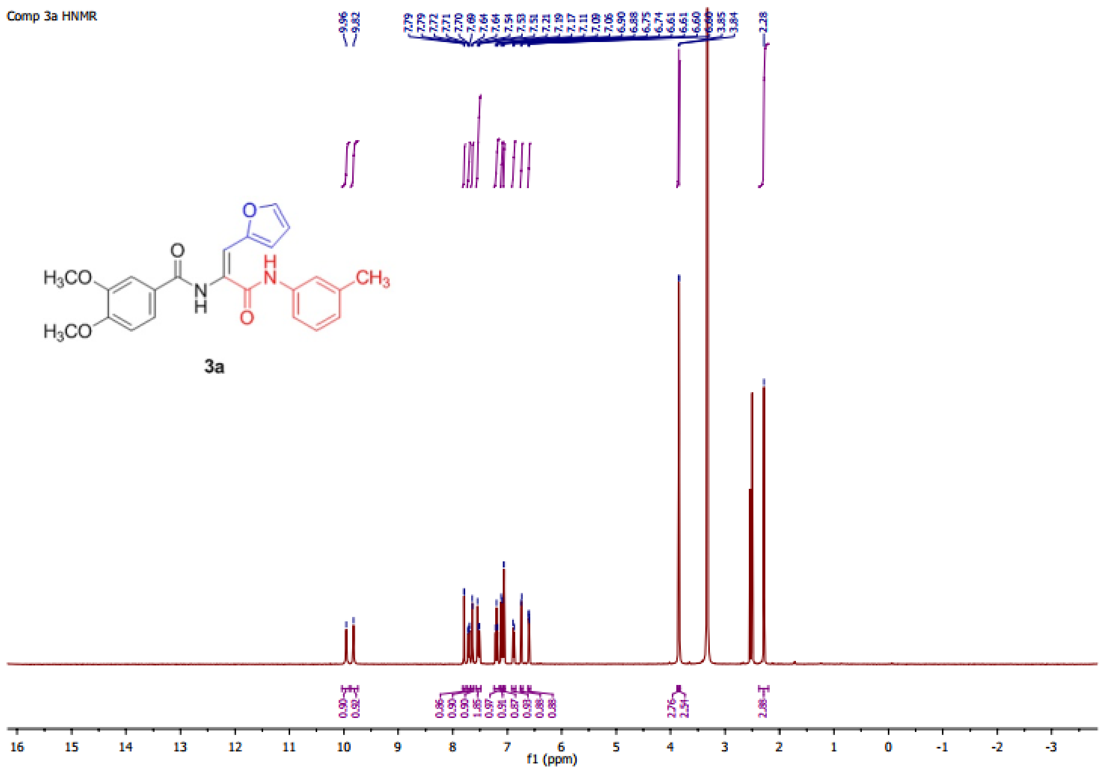


**Figure S4:** $^{1}$H-NMR spectrum of compound **3a.**

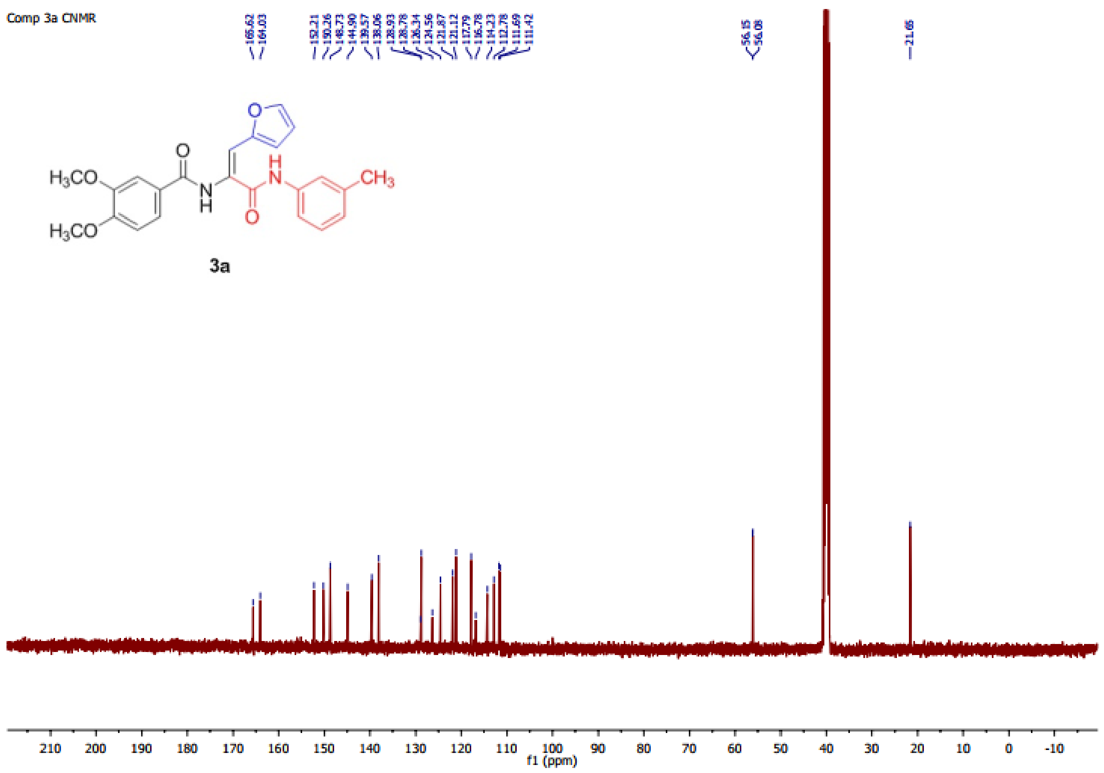


**Figure S5:** $^{13}$C-NMR spectrum of compound **3a.**

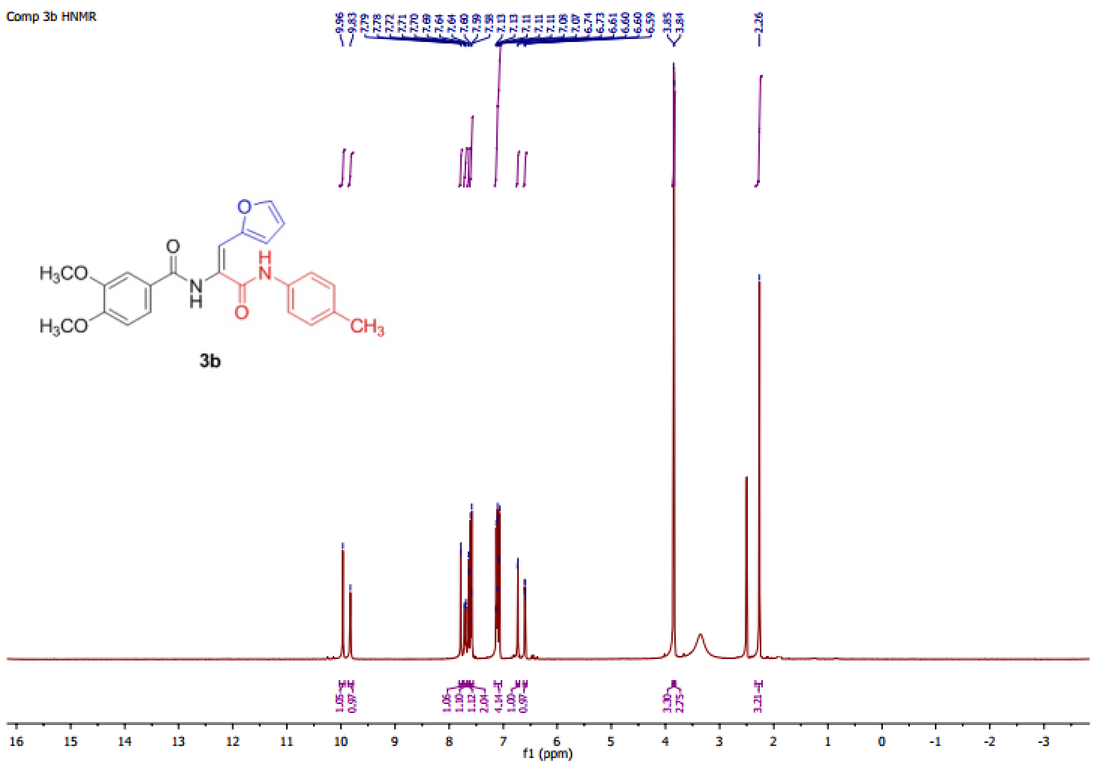

**Figure S6:** $^{1}$H-NMR spectrum of compound **3b.**

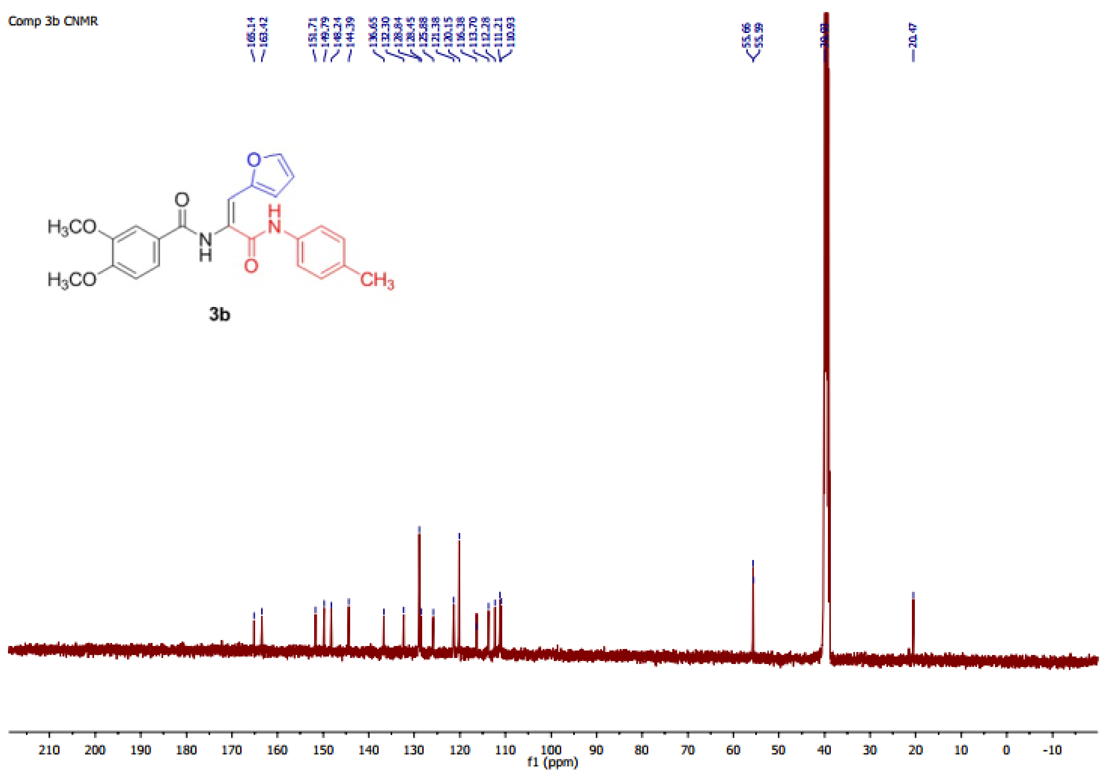


**Figure S7:** $^{13}$C-NMR spectrum of compound **3b.**

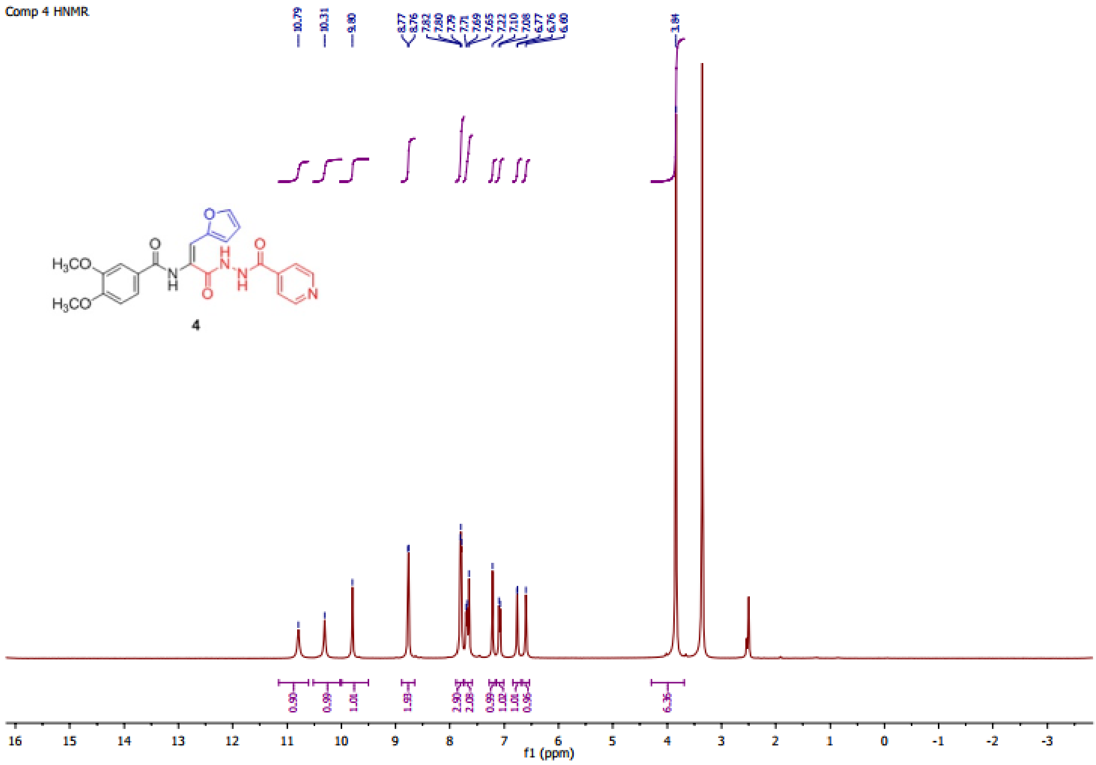

**Figure S8:** $^1$H-NMR spectrum of compound **4.**

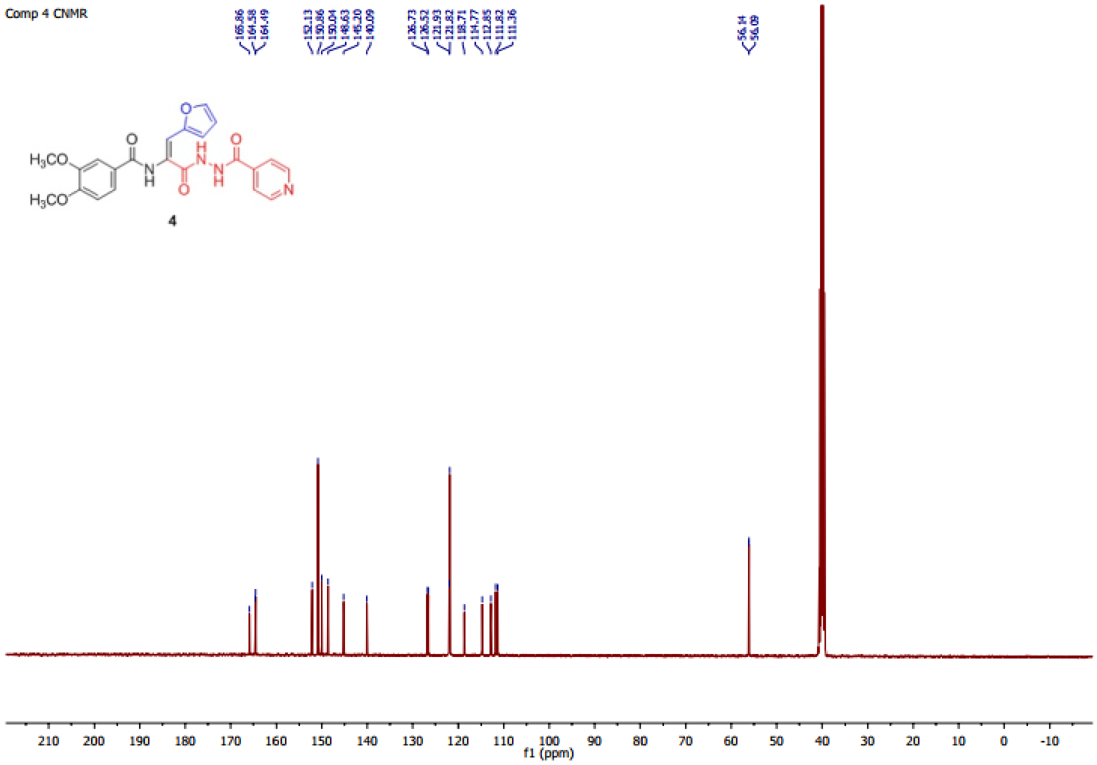

**Figure S9:** $^{13}$C-NMR spectrum of compound **4.**

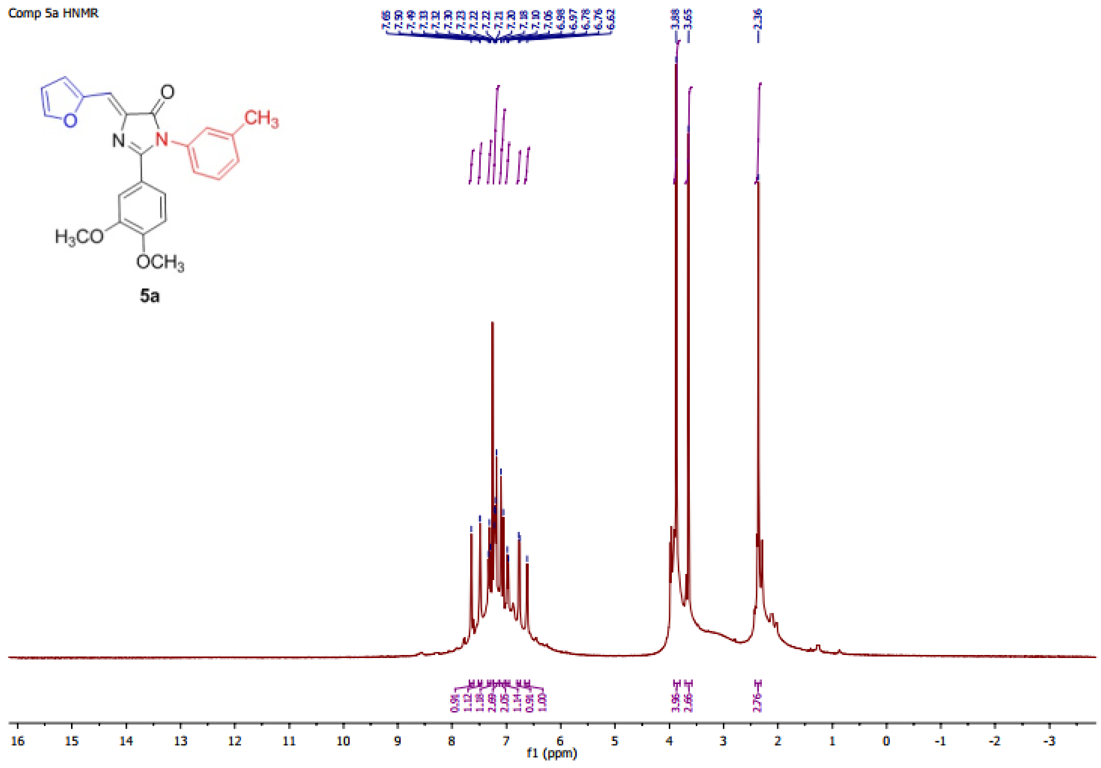


**Figure S10:** $^1$H-NMR spectrum of compound **5a.**

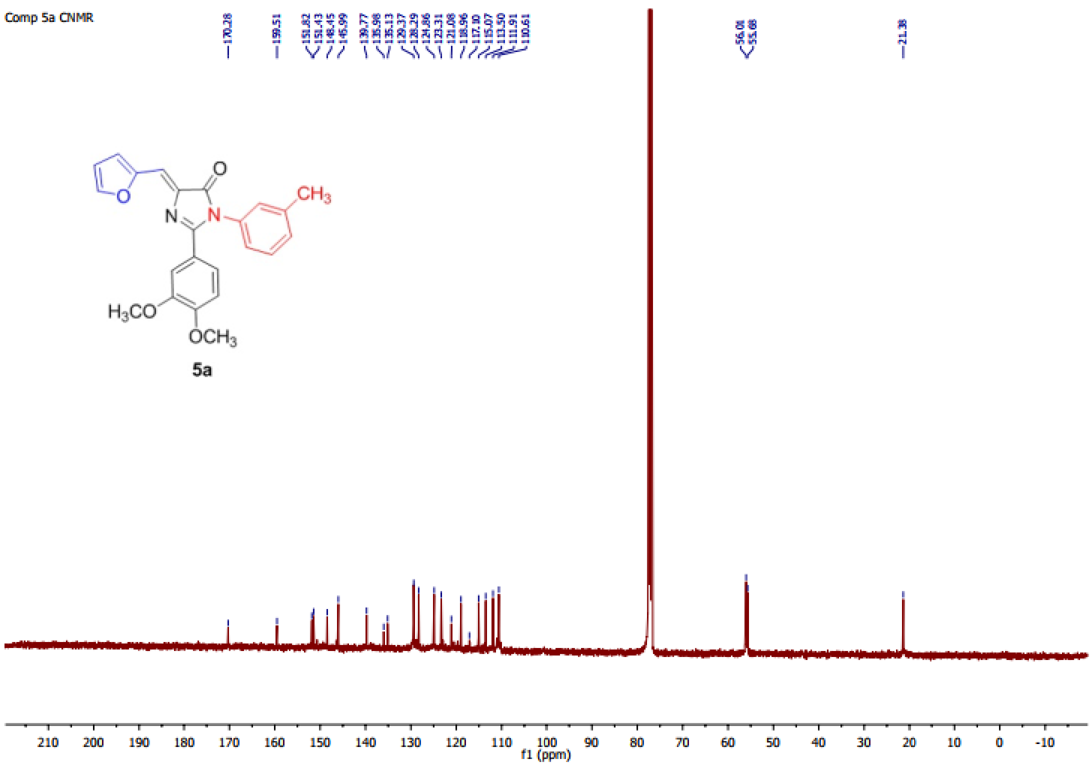


**Figure S11:** $^{13}C$-NMR spectrum of compound **5a.**

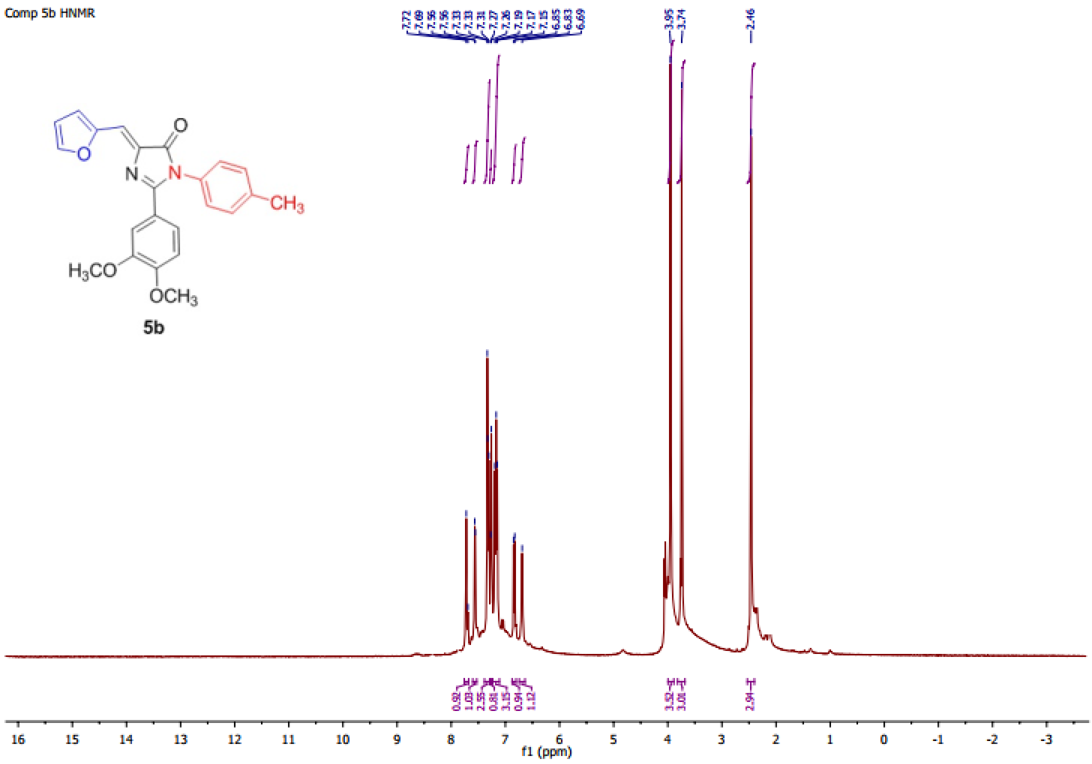


**Figure S12:** $^1$H-NMR spectrum of compound **5b.**

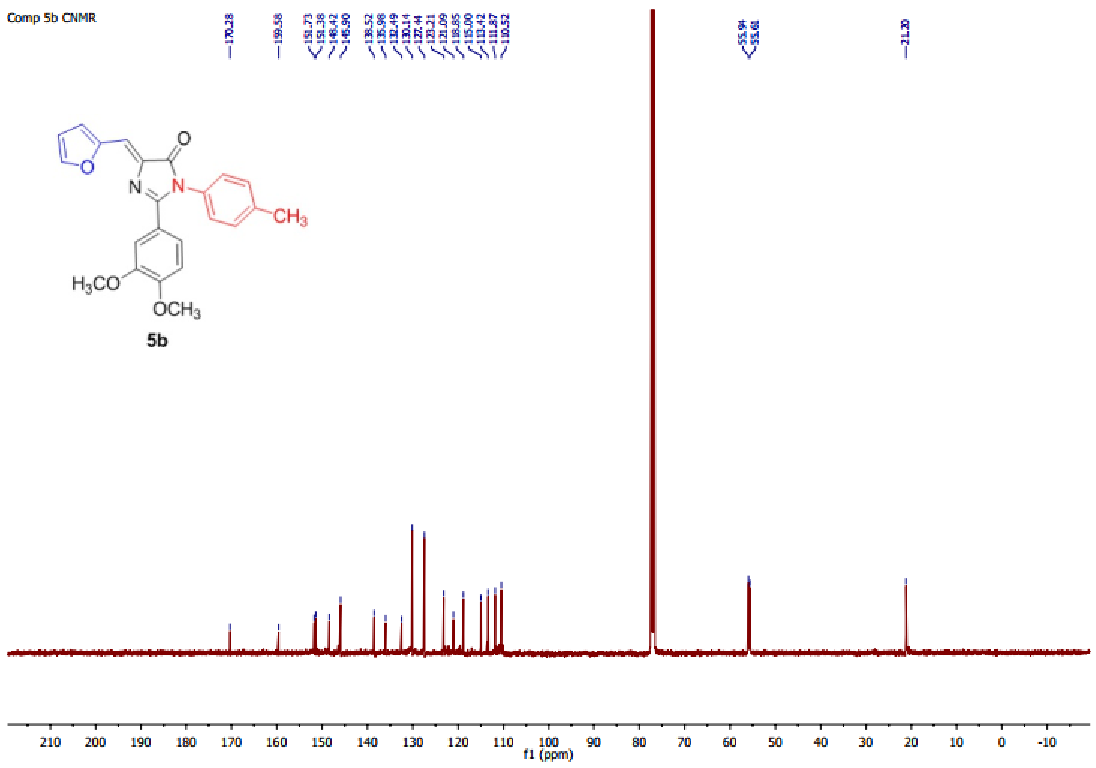


**Figure S13:** $^{13}C$-NMR spectrum of compound **5b.**

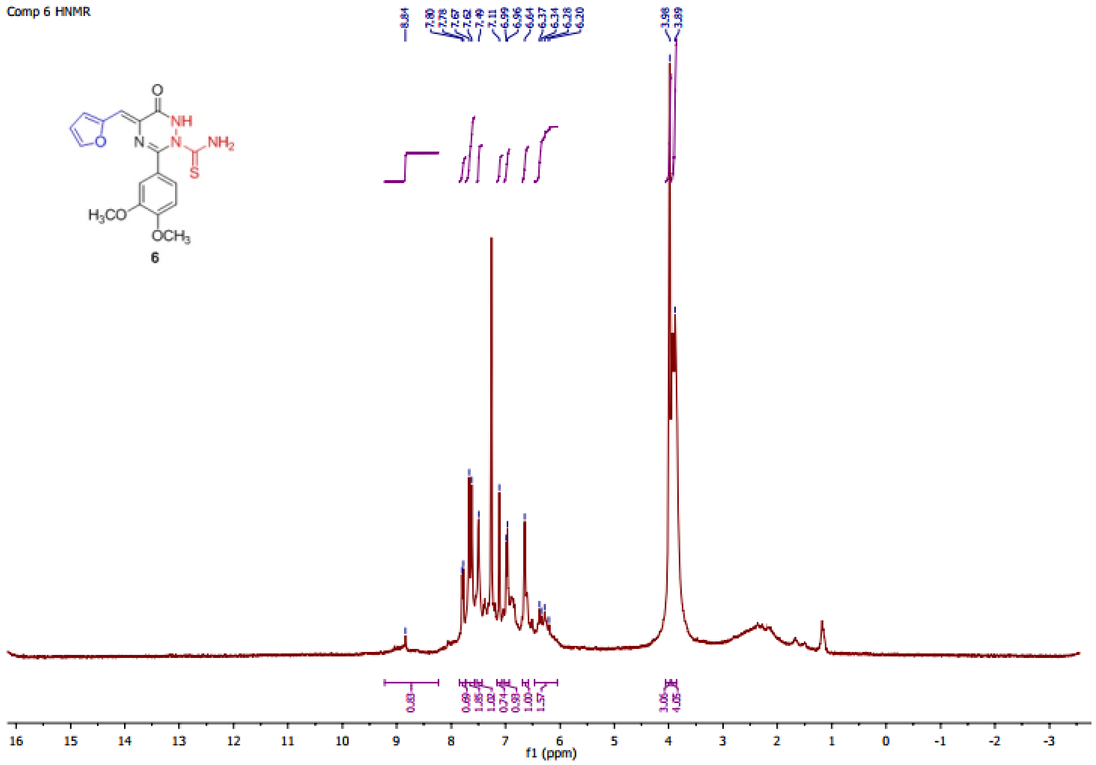


**Figure S14:** $^{1}$H-NMR spectrum of compound **6.**

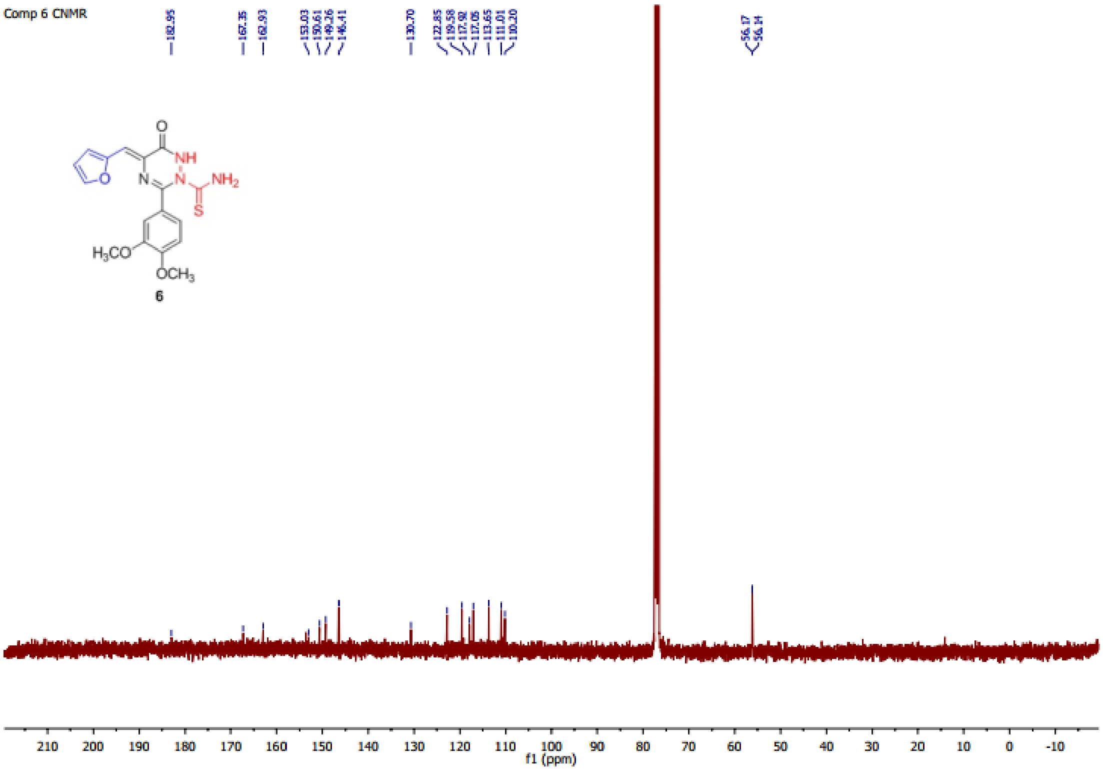


**Figure S15:** $^{13}$C-NMR spectrum of compound **6.**

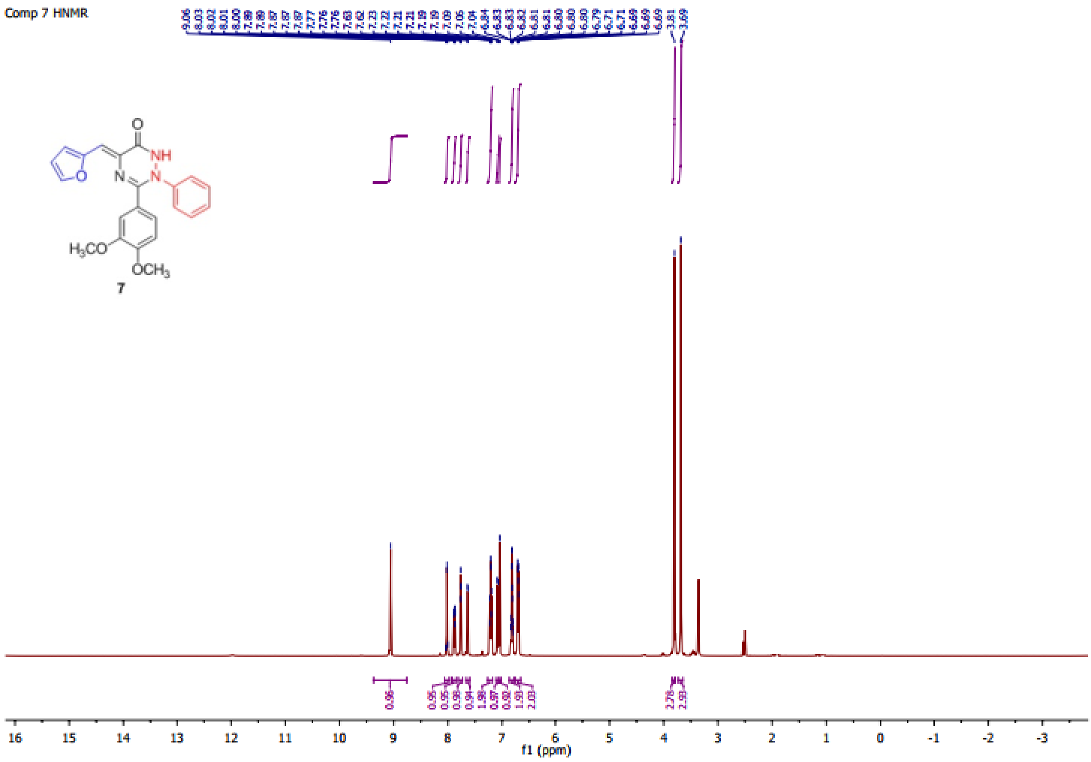

**Figure S16:** $^{1}$H-NMR spectrum of compound **7.**

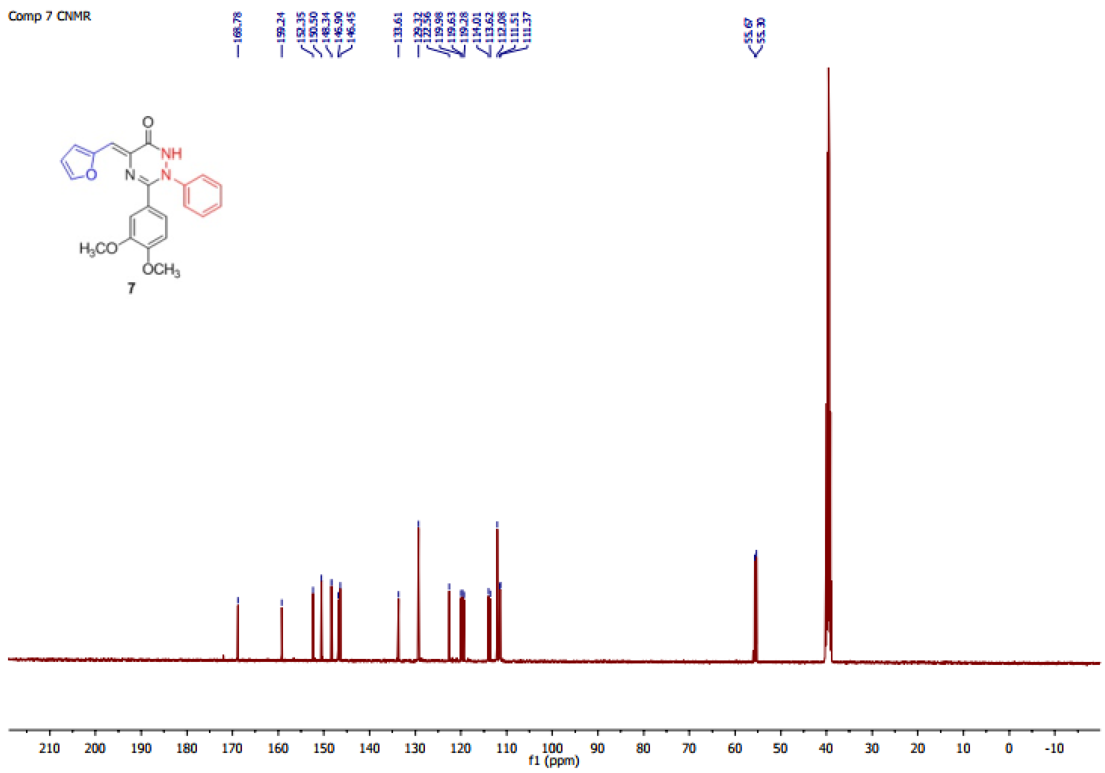


**Figure S17:** $^{13}C$-NMR spectrum of compound **7.**
